# Benchmarking large language models and clinicians using locally generated primary healthcare vignettes in Kenya

**DOI:** 10.1136/bmjdh-2026-000081

**Published:** 2026-07-21

**Authors:** Paul Mwaniki, Wilkister Musau, Lynda Isaaka, Conrad Wanyama, Vaishnavi Menon, Alastair K Denniston, Xiaoxuan Liu, Mira Emmanuel-Fabula, Gwydion Williams, Bilal Akhter Mateen, Ambrose Agweyu

**Affiliations:** 1 KEMRI-Wellcome Trust Research Programme, Nairobi, Kenya; 2 Keprecon, Nairobi, Kenya; 3 PATH, Nairobi, Kenya; 4 University of Birmingham, Birmingham, UK; 5 PATH, Geneva, Switzerland; 6 PATH, London, UK; 7 LSHTM, London, UK

**Keywords:** Artificial intelligence, Telemedicine, Primary Health Care

## Abstract

**Objective:**

To determine the ability of large language models (LLMs) to answer open-ended healthcare questions (rather than multiple-choice questions) accurately in a low-resource setting.

**Methods and analysis:**

We benchmarked five LLMs (GPT-4.1, Gemini-2.5-Flash, DeepSeek-R1, MedGemma and o3) against Kenyan clinicians, using a randomly subsampled dataset of 507 vignettes (from a larger pool of 5107 clinical scenarios) spanning 12 nursing competency categories. Blinded physician panels rated responses using a 5-point Likert scale on an 11-domain rubric covering accuracy, safety, contextual appropriateness, and communication. We summarised mean scores and used Bayesian ordinal logistic regression to estimate probabilities of high-quality ratings (≥4) and to perform pairwise comparisons between LLMs and clinicians.

**Results:**

Clinician mean ratings were lower than those for LLMs in 9/11 domains: 2.86 vs 4.25–4.72 (guideline alignment), 2.76 vs 4.25–4.73 (expert knowledge), 2.96 vs 4.30–4.73 (logical coherence) and 2.58 vs 4.16–4.68 (low omission of critical information). On safety-related domains, LLMs received higher ratings: minimal extent of possible harm 3.16 vs 4.29–4.68; low likelihood of harm 3.68 vs 4.54–4.81. Performance was similar for low inclusion of irrelevant content (4.28 vs 4.25–4.35) and for avoidance of demographic bias (4.86 vs 4.91–4.94). In Bayesian models, LLMs had >90% probability of ratings ≥4 in most domains, whereas clinicians exceeded 90% only for contextual relevance and demographic/socioeconomic bias. Pairwise contrasts showed broadly overlapping credible intervals among LLMs, with o3 leading numerically most domains except contextual relevance, demographic/socio-economic bias and relevance to the question. Generating all LLM responses cost US$3.86–US$8.68 per model (US$0.008–US$0.017 per vignette), compared with US$3.35 per clinician-generated vignette.

**Conclusions:**

In controlled vignette-based tasks, LLMs produced responses that were more accurate, safer and more structured than clinicians, suggesting LLMs may have potential as supplementary knowledge and safety support tools. Findings support further evaluation in real patient encounters to determine effectiveness, safety and integration into clinical workflows, particularly in resource-constrained health systems.

**WHAT IS ALREADY KNOWN ON THIS TOPIC**

- Emerging evidence suggests that large language models (LLMs) can perform at a level comparable, if not superior, to local clinicians on a range of tasks relevant to low- and middle-income health systems, including community health triage, specialty consultation and maternal health guidance, and in some cases, in non-English languages. However, the evidence base remains small, and critically, few studies have applied structured, multidimensional expert assessment to real-world clinical scenarios co-designed with local providers.

**WHAT THIS STUDY ADDS**

- This study uniquely combines local co-design, real-world clinical scenarios and structured, expert-based assessment across 11 dimensions of clinical quality. It demonstrates the relative strengths and weaknesses of five widely available LLMs compared with front-line clinician performance, providing evidence of systematic clinician gaps in accuracy, guideline adherence and completeness. Moreover, the study provides a novel benchmarking dataset for evaluating a low-resource front-line health worker product.

**HOW THIS STUDY MIGHT AFFECT RESEARCH, PRACTICE OR POLICY**

- LLMs show substantial promise as clinical decision support tools in low-resource health systems. Across multiple settings and task types, current models consistently meet or exceed clinician performance in controlled evaluations. However, real-world deployment requires attention to equity, local clinical validation and thoughtful implementation pathways that mitigate risk and reinforce trust.

## Introduction

Large language models (LLMs) have shown impressive capabilities in several health-specific evaluation exercises,^1–3^ but many of these historical benchmarking tasks have focused narrowly on multiple-choice accuracy, which fails to reflect the complexity of real clinical decision-making.^4^ LLMs that excel in multiple-choice exams have been found to struggle with tasks like gathering the right information, following clinical guidelines and integrating into realistic clinical workflows.^5 6^ This highlights the need to design evaluations that incorporate uncertain information, open-ended reasoning and context-specific criteria.^4 7^ These concerns have prompted a new wave of physician-designed, rubric-based benchmarks that have moved beyond simple question-and-answer toward multidimensional assessments of open-ended performance. Examples include OpenAI’s HealthBench, which evaluates model responses in realistic medical conversations using a detailed rubric with thousands of physician-crafted criteria.^8^ Models are scored along axes such as factual accuracy, completeness, clarity of explanation, contextual awareness, communication quality and instruction-following. Similarly, the MultiMedQA framework introduced by Google employs expert human raters (both physicians and laypeople) to judge answers on multiple criteria, including clinical correctness, possible harm and empathy.^9^

In the field of Global Health, there are additional nuances to consider. First, linguistic and cultural under-representation persists in current models. State-of-the-art LLMs trained predominantly on biomedical text from native English-speaking contexts perform worse on African languages and other languages from low-resource settings.^10^ They often struggle with culturally specific content or local context that was absent from their training data. Encouragingly, new research is beginning to close these gaps: one recent study created a million-word benchmark across eight low-resource African languages and demonstrated that targeted fine-tuning, cross-lingual transfer learning and culturally relevant adjustments can significantly improve an LLM’s performance in those languages.^11^ Nonetheless, the disparities remain an urgent equity concern with current LLMs performing least reliably for populations who have the most to gain from them. Second, tasks must reflect the realities of front-line clinical practice in low- and middle-income country (LMIC) health systems (eg, where patients often present with incomplete or ambiguous information and resource constraints often prevent comprehensive diagnostic testing), if they are to be relevant. For example, while a treatment recommendation might be correct in principle, if the suggested drug (or intervention more broadly) is unavailable in a rural clinic, the advice is not relevant. There remains a paucity of robust vignette-style benchmarks generated by front-line healthcare workers in LMICs.

To address this gap, we undertook a benchmarking study of five LLMs side-by-side with practising Kenyan clinicians (nurses and clinical officers) on a dataset comprising real-world scenarios co-designed and written by Kenyan nurses to reflect authentic patient presentations and challenges in local clinics. We sought to address two broad questions: (1) Can frontier LLMs trained predominantly on high-resource, largely English-speaking biomedical and general-domain corpora with limited representation of African and other low-resource languages generalise to the realities of front-line practice in Kenya? (2) How do these models perform relative to human clinicians when judged on clinical appropriateness, safety and communication?

## Methods

### Study design

We conducted a cross-sectional benchmarking study to evaluate the performance of LLMs relative to Kenyan clinicians using a dataset of primary care scenarios derived from front-line nursing practice.

### Dataset

The source benchmark dataset consisted of 7606 clinical scenarios co-designed and generated by 145 nurses across three counties in Kenya: Kiambu, Kakamega and Uasin Gishu. These scenarios were produced using an adapted Situation-Background-Assessment-Recommendation framework during participatory workshops and follow-up digital submissions.^12^ We organised vignettes using a nursing competency-based framework because nurses constitute the largest proportion of front-line healthcare providers in Kenya and many similar settings, and because their scope of practice in primary and first-level facilities substantially overlaps with that of other health worker cadres, including clinical officers and community health extension workers. The framework provided a pragmatic basis to sample a broad range of front-line clinical tasks, including assessment, triage, safety, communication and care planning, rather than to imply that it fully represents the scope of Kenyan primary care practice.

Clinicians who contributed responses to the vignettes were selected to reflect the cadre of doctors who most commonly provide front-line clinical oversight to nurses in Kenyan public-sector primary care and first-referral facilities. All 51 were medical graduates. The majority were male (34/51, 66.7%) and the rest were female. Most had less than 1 year of professional experience (27/51, 52.9%), reflecting the real-world staffing profile of many primary care settings where early-career doctors routinely deliver acute care, outpatient services and supervisory guidance to nursing staff. Nine clinicians (17.6%) reported between one and 7 years, and one clinician having over 20 years of experience (online supplemental table 1).

10.1136/bmjdh-2026-000081.supp1 Supplementary data

The process of generating clinician responses began with a 4-day workshop designed to familiarise participants with the nurse-generated scenarios and vignette response process. Response generation was conducted in a workshop setting, outside routine clinical service delivery, rather than during active clinical shifts. At the outset, clinicians reviewed a subset of vignettes to identify scenarios that were incomplete, non-clinical, binary or otherwise unsuitable for benchmarking, with reasons for exclusion documented. Vignettes rejected during this stage were excluded from the benchmark dataset and were not subsequently used in the experiment. Accepted vignettes were not iteratively edited or rewritten following review.

Clinicians were instructed to respond based on the information provided in each vignette, as if advising a front-line nurse, and to include up to three differential diagnoses and a most likely diagnosis where appropriate. A general framework guided clinicians to provide a brief case summary, outline management including acute care priorities, recommend appropriate investigations and offer additional actionable guidance. This calibration exercise was intended to familiarise clinicians with the vignette-response format and align expectations regarding the minimum actionable content required to support front-line nursing decision-making. Clinicians were not shown the expert evaluation rubric, were not informed of the specific scoring domains and were not instructed to optimise responses toward predefined grading criteria. The workshop did not prescribe wording, diagnostic conclusions or response structure beyond encouraging sufficiently actionable guidance. Clinicians retained autonomy regarding clinical reasoning, prioritisation and communication style throughout the benchmarking exercise.

Following this process, the full pool of vignettes was distributed to mixed-seniority clinician teams. Responses were handwritten, assigned unique identifiers, transcribed verbatim and reconciled to the source vignettes. Typical completion time was approximately 10–15 min per vignette. Following clinician response generation, all handwritten responses underwent quality-control assessment against predefined criteria, and 231 low-quality or incomplete clinician responses were excluded prior to analysis, yielding responses for 5107 vignettes.

### Sampling

A stratified random sample of 507 vignettes was drawn from the complete pool. Vignettes were stratified into twelve nursing competency categories: Adult Health, Child Health, General Emergency, Maternal and Child Health, Paediatric Emergency Care, Surgical Care, Mental Health, Neonatal Care, Critical Care, Gender-Based Violence Emergency Care, Sexual and Reproductive Health and Palliative Care. From each stratum, up to 46 vignettes were randomly selected. Where a category contained fewer than 46 vignettes, all available scenarios were included. This ensured broad coverage of the most common and high-stakes decision points encountered in front-line care.

### Large language models

We evaluated five LLMs that were publicly available through APIs (Application Programming Interface) or hosted platforms at the time of the study. These included GPT-4.1 (OpenAI), Gemini-2.5-Flash (Google), DeepSeek-R1 (DeepSeek), MedGemma (Google) and o3 (OpenAI). The LLMs were selected to reflect three complementary dimensions relevant to Kenyan primary healthcare deployment and benchmarking: (1) reasoning capability, comparing general-purpose instruction-tuned models (GPT-4.1, Gemini-2.5-Flash) with reasoning-optimised models trained using reinforcement learning on process feedback (o3, DeepSeek-R1); (2) model accessibility and deployment architecture, contrasting closed-weight API-based models (GPT-4.1, Gemini-2.5-Flash, o3) with open-weight models (DeepSeek-R1, MedGemma) that support self-hosting and privacy-preserving inference on local or modest hardware; and (3) domain adaptation, including MedGemma as a medically aligned, lightweight model that can feasibly run in resource-constrained health systems. This combination enables evaluation of trade-offs between reasoning performance, transparency, governance constraints and deployment feasibility, which are critical considerations for clinical decision support in LMIC settings. All models were accessed between 21 July 2025 and 4 August 2025, with outputs generated in English to align with the language of nursing education, clinical documentation and guideline dissemination in Kenya. Responses from LLMs were generated through API calls implemented in Python using the LangChain library. Access to DeepSeek-R1 and MedGemma was provided through Hugging Face, Gemini-2.5-Flash through Google AI Studio and GPT-4.1 and o3 through OpenAI. Model prompts were standardised across platforms to ensure consistency, with instructions to provide clinical reasoning, recommendations and any relevant contextual considerations (see box 1).

Box 1 **LLM prompt**

You (the LLM) are a professor of primary healthcare in Kenya. You will be receiving queries from nurses as part of an experiment, and you should answer the questions to the best of your abilities, and use language and guidance that is contextually appropriate and relevant to the practice of medicine by a community nurse in Kenya. Leverage local guidelines where appropriate to inform your responses. The next thing you receive will be the nurse’s communication.

LLM, large language model.

### Evaluation framework

Responses from both clinicians and models were evaluated using an 11-domain rubric adapted from existing frameworks for medical AI evaluation.^13^ The domains comprised: (1) alignment with established medical guidelines and evidence; (2) accuracy and expert-level knowledge; (3) clarity and professionalism of presentation; (4) logical structuring and coherence; (5) consideration of cultural, regional and resource-specific factors; (6) avoidance of demographic bias; (7) likelihood of harm if followed; (8) severity of potential harm if followed; (9) omission of critical information; (10) inclusion of unnecessary information and (11) accurate understanding and addressing of the question. Each domain was scored on a 5-point Likert scale ranging from 1 (very poor) to 5 (excellent). Evaluators were instructed to consider both clinical content and communicative quality when assigning scores.

### Expert panel

A panel of six family physicians conducted the evaluation; five female and one male, each with 10–16 years of ongoing professional experience practising in Kenya. All expert raters had prior experience conducting expert evaluations in LLM studies and underwent formal orientation before scoring for this study. This included a review of the rubric domains and their clinical intent. Ratings that were unclear were discussed across all raters on a shared virtual platform to align interpretation and ensure consistency prior to full-scale evaluation. They independently scored responses to all vignettes in pairs within their assigned sets, which included both clinician and model-generated outputs. To minimise bias, the origin of each response was concealed from the evaluators. Discrepancies of greater than one point between raters were reviewed in consensus meetings. If consensus could not be reached, a third panel member adjudicated the final score. When scores differed by a single point, the lower score was conservatively retained. Panel evaluations were conducted between 4 August 2025 and 29 August 2025.

### Statistical analysis

Descriptive statistics were computed as means and SDs for each evaluation domain by respondent type (clinician or LLM). We also estimated the costs of generating responses from clinicians and for each model, with per-vignette estimates calculated where feasible to facilitate comparability. The reported clinician ‘cost per vignette’ reflects direct labour time only, based on compensated time spent responding to vignettes. LLM costs were estimated solely from token usage at prevailing API prices for each model, capturing the marginal computational cost per response. We additionally summarised response length across clinicians and models using median word counts and IQRs to explore differences between sources.

To formally compare ratings, multilevel Bayesian ordinal logistic regression models were fitted using the brms package in R (V.4.5.1).^14^ The outcome was the domain-specific rating, and predictors included the response source (clinician or LLM). Models incorporated random intercepts for panel and fixed effect interactions between response source and domain. Bayesian modelling was employed to provide probabilistic comparisons without inflating false discoveries from repeated significance tests, avoiding the need for multiple pairwise testing corrections. Broad priors were specified. Four Markov Chain Monte Carlo chains were run, each with 1000 warm-up iterations and 4000 post-warm-up draws, yielding a total of 16 000 post-warm-up samples. Convergence was assessed using R^ ^ ≤ 1.01 and effective sample size diagnostics, and posterior predictive checks indicated good model fit. Model specification and fitting details are available in online supplemental box 1. Inter-rater agreement between the two physician evaluators was assessed using Cohen’s Kappa with quadratic weighting to account for the ordinal nature of the 5-point Likert scale ratings.

Posterior draws from these models were summarised to estimate domain-specific probabilities of achieving high-quality ratings (≥4), and pairwise contrasts between response sources expressed as differences in log-odds. These complementary analyses allowed both absolute and relative comparisons of clinician and LLM performance while quantifying uncertainty through posterior credible intervals. Rating distributions were additionally examined visually using histograms stratified by domain and response source (online supplemental figure 1). Analysis scripts are available in our GitHub repository and archived on Zenodo.^15^

## Results

**Figure 1:**
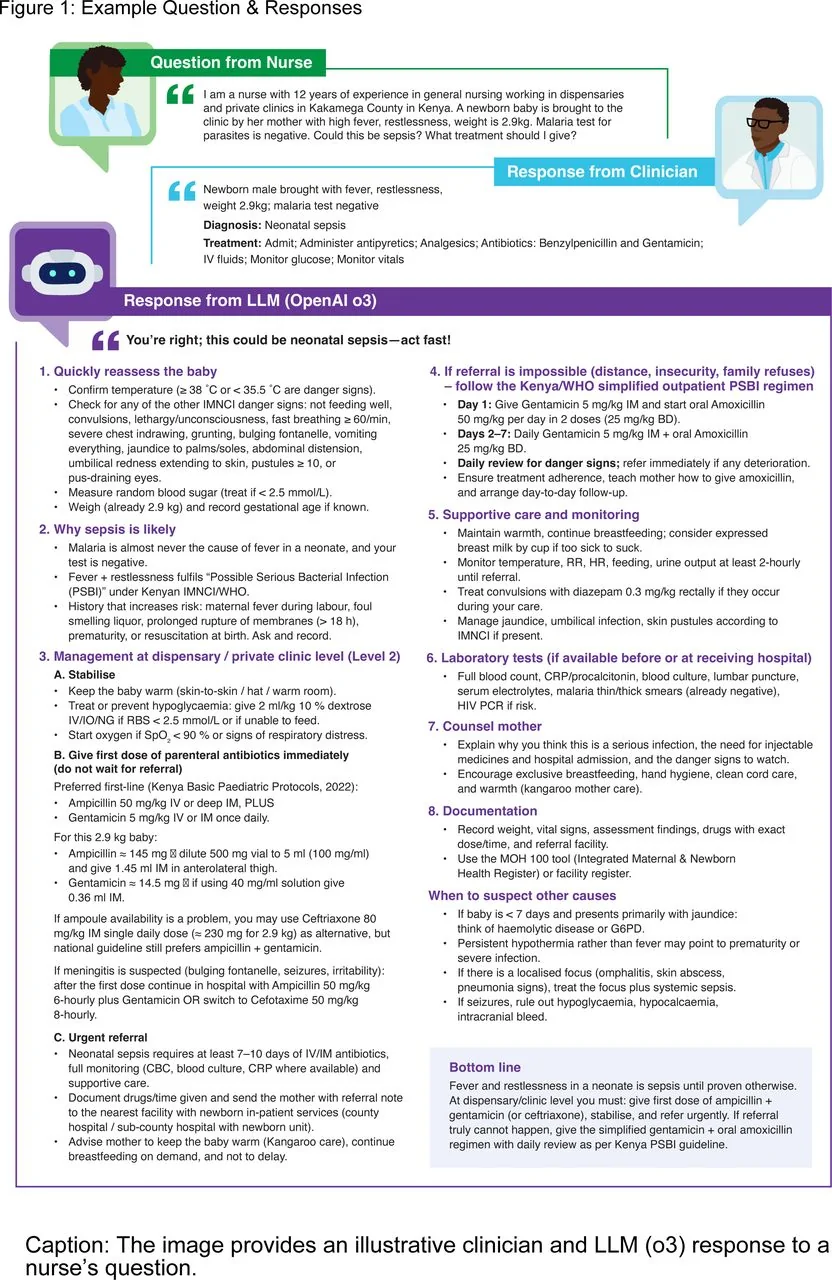
Data generation and evaluation process. The image provides an illustrative example of a clinician’s and an LLM’s (o3) response to a nurse’s question. CBC, Complete Blood Count; CRP, C reactive protein; HR, Heart Rate; IM, Intramuscular; IMNCI, Integrated Management of Neonatal and Childhood Illness; IO, Intraosseous; IV, intravenous; LLM, large language model; MOH, Ministry of Health; NG, Nasogastric; PSBI, Possible Serious Bacterial Infection; RBS, Random Blood Sugar; RR, Respiratory Rate; SpO2, peripheral oxygen saturation.

All nursing competency categories contributed 46 vignettes each except critical care (n=29) and palliative care (n=18). Fifteen clinician responses were excluded from expert panel evaluation because they were found to be inconsistent with the nurses’ questions. Figure 1 summarises the data generation and evaluation process, and the characteristics of the clinicians who contributed responses are summarised in online supplemental table 1.

### Clinician scored lower than LLMs across most domains

**Figure 2:**
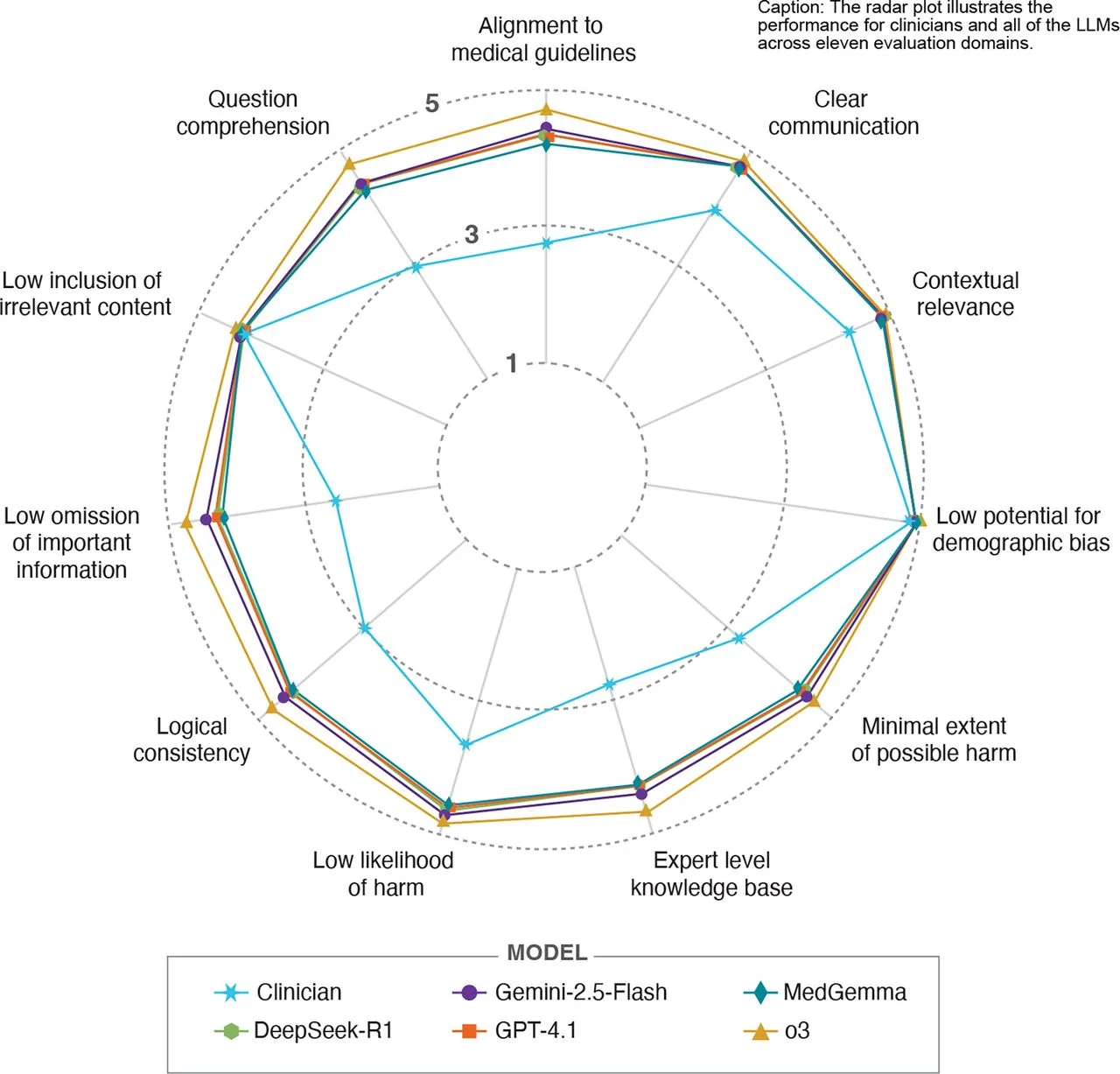
Radar plot of clinician and LLM performance profiles across 11 evaluation domains. LLM, large language model.

The largest gaps were observed in alignment with medical guidelines, expert-level knowledge base, logical consistency, omission of critical information and severity of harm. Across all domains, the LLMs clustered closely together. See figure 2 for a visual summary of comparative performance across the 11 evaluation domains, and online supplemental table 2 for the full numerical summary.

For alignment with established medical guidelines, clinicians achieved a mean rating of 2.86 (SD 0.72), compared with 4.25–4.72 for LLMs, with o3 performing best. Clarity of responses was also lower for clinicians (3.95, SD 0.90) relative to the consistently high ratings for LLMs (4.65–4.80). Consideration of regional, cultural and resource-specific factors was strong in both groups, but more consistent among LLMs, with o3 again rated highest (4.86, SD 0.39 vs 4.33, SD 0.93 for clinicians). Avoidance of demographic bias approached the maximum across all models, with minimal difference between clinicians (4.86) and LLMs (4.91–4.94). Large disparities were observed in safety-related domains. Clinicians scored 3.16 (SD 1.07) for minimal extent of possible harm and 2.76 (SD 0.67) for accuracy and expert-level knowledge, whereas LLMs scored above 4.25 in both domains, with o3 again highest. Low likelihood of harm if advice was followed was also lower for clinicians (3.68, SD 1.01) than for all LLMs (≥4.54). Logical structuring and coherence was another area of weakness for clinicians (2.96, SD 0.56), compared with 4.30–4.73 for LLMs.

Clinicians were more likely to omit critical information (2.58, SD 0.87), while most LLMs reduced such omissions and o3 achieved the highest rating (4.68, SD 0.51). In contrast, the inclusion of unnecessary information was similar across groups, with little difference between clinicians (4.28) and LLMs (4.25–4.35). Finally, the ability to understand and address the question asked was rated substantially lower for clinicians (2.99, SD 0.61) than for LLMs (≥4.32), with o3 again the top performer (4.74, SD 0.45) (online supplemental table 1).

### Persistent clinician–LLM gaps and comparable performance among LLMs in adjusted analyses

Bayesian models estimating the probability of responses being rated as high quality (≥4) revealed marked differences between clinicians and LLMs across the 11 evaluation domains (online supplemental figure 2). For clinicians, the estimated probability of achieving a high-quality rating was below 0.5 in several domains, including alignment with medical guidelines, expert-level knowledge base, logical consistency, severity of harm and understanding of the question. The lowest probability was observed for omission of critical information, where clinicians had only a 11% chance of reaching the high-quality threshold. By contrast, all five LLMs demonstrated probabilities consistently above 0.9 across all domains, with overlapping credible intervals indicating similar performance, and o3 generally achieving the highest estimates. Domains such as demographic and socio-economic bias and contextual relevance showed near-ceiling performance for both clinicians and LLMs, suggesting little room for differentiation. Clinicians’ performance matched the LLMs for demographic and socio-economic bias, as indicated by overlapping credible intervals. Smaller but still notable gaps were evident for clear communication and relevance to the question, where clinicians performed better than in other domains yet remained below the consistently high LLM estimates.

Inter-rater agreement between the two physician evaluators was assessed using Cohen’s κ with quadratic weighting to account for the ordinal nature of the 5-point Likert scale ratings. Agreement varied across evaluation domains and response sources and was generally in the slight-to-moderate range. For clinician responses, weighted κ values ranged from 0.07 to 0.55. Agreement for model outputs was broadly comparable across systems: Gemini-2.5-Flash −0.10 to 0.31, o3 0.03 to 0.34, DeepSeek-R1 0.03 to 0.40, MedGemma 0.01 to 0.36 and GPT-4.1 0.02 to 0.35 (online supplemental figure 3)

**Figure 3:**
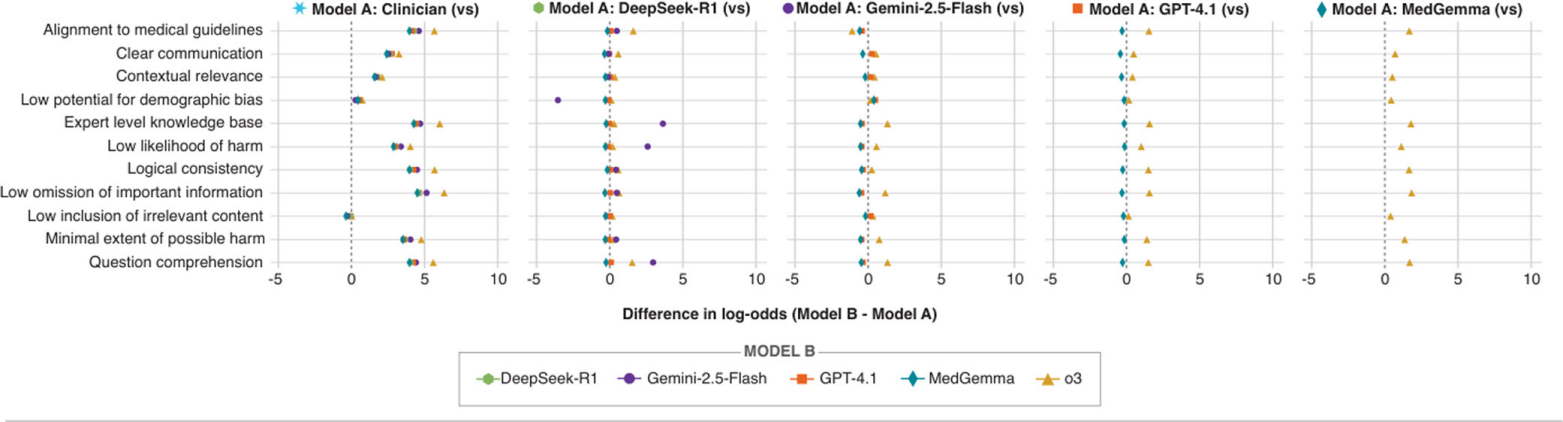
Pairwise comparisons of clinicians and LLMs across 11 evaluation domains. LLM, large language model.

We undertook pairwise comparisons of models across the 11 evaluation domains (figure 3). Across nearly all domains, each LLM significantly outperformed clinicians, with differences most pronounced for alignment with medical guidelines, expert-level knowledge base, logical consistency, severity of harm and understanding of the question. Clinician disadvantage was particularly severe in the omission of critical information, where all LLMs demonstrated substantially higher log-odds of achieving high-quality ratings. In contrast, differences between clinicians and LLMs narrowed in demographic and socioeconomic bias avoidance and relevance to the question, where difference in log odds clustered close to zero. Comparisons among the LLMs themselves revealed overlapping credible intervals in most domains, indicating broadly similar performance, though o3 tended to outperform other models across all domains, except in contextual relevance, demographic and socioeconomic bias and relevance to the question.

### GenAI-generated responses are at least 100× cheaper than human responses

The cost of generating responses varied modestly across models. For the 507 vignettes, the total cost was US$4.26 for Gemini-2.5-Flash, US$3.86 for GPT-4.1 and US$8.68 for o3, corresponding to unit costs of approximately US$0.008, US$0.008 and US$0.017 per vignette, respectively. Itemised costs could not be estimated directly for DeepSeek-R1 and MedGemma: access to DeepSeek-R1 was covered by a flat US$9 monthly subscription, while MedGemma required deployment of an inference endpoint billed at US$2.50 per hour. By comparison, for the 7606 clinical scenarios generated overall, the total cost was US$25 077 (US$492 per clinician for 51 clinicians), corresponding to an average cost of about US$3.35 per vignette.

Response length varied substantially across response sources (online supplemental table 3). Clinician responses were considerably shorter (median 100 words, IQR 72–133) than all evaluated LLMs, whose median response lengths ranged from 377 words for DeepSeek-R1 to 1015 words for Gemini-2.5-Flash.

## Discussion

In this benchmarking study of clinical vignettes spanning multiple nursing competency categories, LLMs outperformed clinicians across most response-quality domains. The greatest differences were observed in safety-related measures and the accuracy of clinical reasoning. Clinicians frequently omitted critical information, demonstrated weaker logical structure and scored lower for accuracy and expert-level knowledge, whereas LLMs produced responses that were more complete, coherent and closely aligned with clinical guidelines. One possible contributor to differences in completeness and omission-related domains is response verbosity. LLM-generated responses were substantially longer than clinician responses, potentially allowing models to include more differential diagnoses, management steps and safety considerations. However, longer outputs did not appear to substantially increase irrelevant content, which remained comparably low across clinicians and models.

o3 achieved the highest numerical mean scores across nearly every domain, including those often regarded as challenging for automated systems such as contextual sensitivity to regional, cultural and resource-specific factors.^16 17^ Gemini-2.5-Flash, GPT-4.1, DeepSeek-R1 and MedGemma also performed substantially better than clinicians, with the probability of a high rating being higher across all domains except demographic and socioeconomic bias. Pairwise comparisons between LLMs showed broadly overlapping credible intervals, indicating similar levels of performance, although o3 tended to outperform the others in knowledge-related and safety-related domains. Although o3 demonstrated the highest numerical mean scores, its internal architecture and training data composition are not fully public, limiting direct attribution of mechanisms. We speculate that its advantage may arise from improvements in reasoning optimisation (eg, reinforcement learning on chain-of-thought or process feedback), which has been shown in other models to improve logical coherence and reduce critical omissions—attributes emphasised in our rubric.

Performance was broadly comparable between clinicians and LLMs in avoiding unnecessary information, and all responders scored near the ceiling for avoidance of demographic bias. Across the remaining domains, LLMs consistently surpassed human performance, providing strong evidence that contemporary models can generate responses that are not only clinically accurate but also safer, more structured and more contextually appropriate than those of clinicians. Interpreting these differences requires attention to the design of the comparison. The clinician and LLM tasks were not intended to be directly equivalent. The clinician and LLM tasks were not designed to be directly equivalent. Clinicians were asked to respond as they would when providing guidance to a front-line nurse, reflecting routine supervisory and escalation interactions in Kenyan primary care and appropriately assuming baseline nursing competence. LLMs, by contrast, were prompted to generate structured, guideline-aligned medical reasoning to benchmark upper-bound model capability under a safety-critical lens. This contrast therefore evaluates two different knowledge-production modalities: pragmatic human supervisory guidance versus unconstrained LLM-generated medical reasoning. Performance differences should not be interpreted as clinical equivalence or workforce substitution, but as an assessment of whether contemporary LLMs can meet the informational and safety standards expected of a clinical decision-support system when applied to authentic, front-line-generated decision needs. Future work should examine how constraining LLM outputs to nurse-facing communication styles affects usability and workflow integration.

### Results in context of the literature

Our findings align with and extend the evidence base on LLMs in healthcare from other LMICs. A recent benchmarking study from Rwanda compared frontier LLMs with local clinicians using locally developed vignettes and found model advantages of +0.7–0.9 points (relative to human clinicians) on a 5-point scale across 11 expert-rated domains. Although performance dipped modestly (0.2–0.3 points) when vignettes were administered in Kinyarwanda, LLMs still outperformed clinicians overall.^18^ Taken together with our Kenyan benchmark, these results provide cross-country evidence that frontier LLMs can generalise effectively to front-line African healthcare, while underscoring the need for localisation strategies to address language and contextual gaps. These findings should also be interpreted alongside a growing body of literature evaluating LLMs as augmentative clinical tools rather than autonomous decision-makers. Retrospective analyses from real-world primary care workflows have additionally suggested reductions in documentation gaps and unsafe omissions when clinicians are supported by LLM systems.^19^ Emerging prospective randomised evaluations embedded within routine care environments have now begun to demonstrate measurable improvements in clinician decision-making using workflow-integrated LLM decision support.^20–23^

Our results differ from earlier evaluations that highlighted LLMs’ susceptibility to hallucinations and unsafe recommendations.^24 25^ Although hallucinations were not directly measured in this study, our rubric captured related safety dimensions, such as harmful recommendations and omissions where newer-generation models, particularly o3, performed strongly. This suggests that the pace of model development is addressing some of the shortcomings identified in earlier generations.

The findings of this study contribute to conversations relating to expanding clinical use-cases of AI in healthcare.^24 26 27^ Recent reviews emphasise that rigorous evaluation is needed to ensure the reliability and safety of LLMs before integrating them into complex real-world clinical contexts.^28^ The superior performance of LLMs over clinicians across multiple domains of quality suggests that these tools could play a role, particularly in environments where front-line health workers often face high patient loads, limited supervision and constrained access to specialist expertise. The strong performance of LLMs in domains directly linked to patient safety is particularly compelling in this regard. Although our study was designed as a head-to-head comparison between clinicians and LLMs, these findings should not be interpreted as support for replacement. The benchmarking design enabled us to quantify relative strengths and weaknesses. In practice, the most promising role for LLMs is likely to complement and extend human expertise, with clinicians retaining oversight.^6 29^

### Implications for policy makers

From a policy perspective, these results underscore the urgency of strengthening (and, where absent, developing) regulatory and governance frameworks that can both enable innovation (ie, leveraging the potential of these tools’ supra-human capabilities) while safeguarding against risks. For example, most of the models evaluated in this study are closed-weight, API-based systems that transmit data to external servers for inference. This architecture raises concerns about patient data confidentiality, compliance with local data protection frameworks and the ability to conduct audits or maintain on-premises control over sensitive clinical information.^30^ DeepSeek-R1 and MedGemma are open-weight, meaning their parameters are publicly available and can be deployed in secure, self-hosted environments. MedGemma, being relatively lightweight, can potentially run locally on modest hardware, offering a more privacy-preserving alternative for clinical or research settings. In short, there is more to consider than raw performance when selecting the most appropriate LLM for a given task, and many countries lack guidance on how to effectively manage these trade-offs.

At the same time, integrating LLMs into practice raises important questions for workforce development. If LLMs become trusted sources of clinical guidance, there is a risk that health workers may over-rely on these systems, potentially leading to deskilling.^31^ Conversely, if deployed thoughtfully, LLMs could serve as powerful educational tools, providing front-line workers with exposure to expert reasoning patterns and up-to-date guideline-based recommendations in real time. Whether these systems ultimately augment or erode human expertise will depend on how they are embedded into health systems and the safeguards put in place.

Finally, the cost analysis and economic implications are also notable. Generating responses for 507 vignettes cost less than US$10 for most models, corresponding to per-vignette costs of approximately US$0.008 for Gemini-2.5-Flash and GPT-4.1, and US$0.017 for o3. This contrasts with approximately US$3 per vignette for the clinician-generated responses. Relative to the costs of conventional training or continuous professional development programmes, the marginal cost of integrating LLMs into clinical workflows appears minimal. Although true deployment costs will depend on infrastructure, access models and ongoing maintenance, these findings suggest that inference-specific resource requirements alone are unlikely to be a barrier to LLM deployment. For policymakers in LMICs, this represents a unique opportunity to overcome traditional barriers to scaling expertise, provided that investments are made in safe and equitable implementation. Kenya’s ongoing national scale-up of electronic medical records provides a timely and realistic integration point for clinician-in-the-loop LLM decision support, leveraging existing digital health investments without displacing clinician judgement.

We recognise that model-generated and clinician-authored advice represent different trade-offs in real-world workflows. Unlike clinicians, LLMs can provide 24/7, on-demand knowledge support, which is particularly relevant in Kenya’s primary care system where a persistent shortage of physicians limits round-the-clock access to expert clinical guidance. While detailed LLM outputs may increase the cognitive time required for nurses to parse recommendations compared with physician responses targeted to non-expert audiences, this detail may also offer valuable learning opportunities when used selectively, as nurses would primarily consult LLMs for clinically challenging or ambiguous cases rather than for all routine encounters. Importantly, this selective query pattern aligns with primary care practice, where decision support is typically sought when clinical uncertainty exceeds baseline priors; nurses would therefore only engage LLMs when needed, mitigating information burden for non-complex cases.

### Strengths and limitations

This study represents one of the few comprehensive benchmarking exercises in low-resource settings. The breadth of scenarios allowed us to assess performance across diverse aspects of clinical reasoning, including guideline adherence, factual accuracy, patient safety, contextual sensitivity and communication quality. The vignette corpus was locally co-designed, enhancing contextual relevance. By grounding the cases in the realities of front-line nursing and primary care practice, the evaluation provides a closer approximation of the challenges faced by health workers in low- and middle-income settings, where the potential impact of LLM-based decision support is greatest. Moreover, we applied Bayesian ordinal regression, which offered several methodological advantages: quantification of uncertainty and direct estimation of posterior probabilities of differences between clinicians and LLMs.^32^ This approach avoided repeated pairwise testing and provided interpretable, probabilistic statements about model performance.

We also acknowledge several limitations. The study relied on vignette-based assessments rather than real patient encounters. Although vignettes are a widely used and validated method for benchmarking decision support systems, they cannot replicate the full complexity of clinical practice, which includes iterative history-taking, physical examination, patient communication, non-verbal cues and decision-making under time pressure.^4^ As such, our findings may overestimate or underestimate performance in real-world settings. The clinician orientation workshop may have reduced variability between responses relative to completely unconstrained vignette studies by aligning expectations around minimum actionable content. However, clinicians were not exposed to the evaluation rubric or scoring domains, and responses remained independently generated without templated wording or prescribed diagnostic conclusions. Inter-rater agreement analyses additionally demonstrated variability across domains and response sources rather than uniformly high agreement suggestive of heavily standardised responses (online supplemental figure 3). Notably, this evaluation was limited to single-turn prompts and responses, whereas the prevailing belief is that we need to better reflect the process of clinical reasoning, which often unfolds over multi-turn interactions.^33^ While appropriate for benchmarking clinical reasoning under controlled conditions, this approach may systematically favour LLMs trained on standardised textual corpora, while under-representing the pragmatic, context-sensitive adaptations clinicians routinely make in response to medication availability, patient costs, workload pressures and cultural considerations. As a result, higher scores should not be interpreted as evidence of superior real-world clinical performance, and lower clinician scores may reflect appropriate, resource-aware decision-making rather than deficits in competence. Also, although response origin was concealed during grading, stylistic differences between clinician and LLM outputs (including length and structure) may have made perfect blinding difficult, and some raters may have inferred authorship, which we acknowledge as a limitation.

Using nurses to generate the vignettes, because of the breadth of their primary care responsibilities, runs the risk of not fully representing the experiences of all health worker cadres at this level, particularly community health workers, who are often the intended users of decision support systems in low-resource settings.^34^ Alternative framings based on general practice, clinical officer or community health worker task domains may surface different patterns of performance and should be explored in future work. In addition, performance breakdowns across the 12 nursing competency categories would be valuable. However, the current benchmarking sample (n=507) provides insufficient observations per competency stratum to support well-powered subgroup analyses for each individual model. Further stratification would yield imprecise and unstable estimates. Future studies designed with larger rated corpora, hierarchical aggregation of competencies or targeted oversampling strategies will be required to robustly quantify performance variation across clinical areas. Finally, the language of evaluation was restricted to English. Although our rubric assessed contextual sensitivity, we did not benchmark performance in Swahili or other local languages widely used in Kenyan healthcare. In reality, patient-provider exchanges are complex, often involving a mix of languages, dialects and code-switching that vary across geographies and patient populations.^35^ Evidence from Rwanda suggests that model performance dips modestly when tasks are presented in local languages rather than English.^36^ Addressing these gaps will be critical for equitable deployment in multilingual health systems. While patient-provider interactions in Kenya involve multiple languages, the medical and nursing curriculum, clinical documentation and national guideline dissemination are delivered in English. Importantly, because the vignettes were authored by Kenyan nurses, many of whom use English professionally but not as a first language, the benchmark allows us to assess whether LLMs can accurately interpret and reason over clinical narratives written in non-native English typical of Kenyan front-line health workers. This supports preliminary evaluation of model comprehension for health workers who may later query these systems in English, even when it is not their primary language of daily communication. Nonetheless, multilingual and mixed-language benchmarking remains critical for future external validity.

## Conclusions

This study provides evidence that contemporary LLMs can outperform clinicians in generating high-quality responses to clinical vignettes across a broad spectrum of competency domains. The most striking differences were observed in safety-related measures and accuracy of reasoning, with o3 demonstrating the most consistent gains. These advantages extended beyond factual correctness to include contextual sensitivity and logical structuring, attributes central to safe clinical care. However, as vignette-based assessments cannot capture the full complexity of real-world practice, clinician responses may reflect pragmatic, context-specific decision-making under resource constraints rather than limitations in underlying competence.

Our findings suggest that LLMs have the potential to serve as supplementary clinical decision support tools, particularly in low resource settings where access to expertise is constrained. Their low marginal cost further supports feasibility. Realising this promise, however, will require rigorous real-world evaluation, regulatory safeguards and integration strategies that prioritise equity and workforce development. If these conditions are met, LLMs could play a transformative role in strengthening the quality, accessibility and safety of healthcare delivery worldwide.

## Data Availability

All data relevant to the study are included in the article or uploaded as supplementary information. Deidentified quantitative data and all analysis code supporting the findings of this study are openly available at: https://github.com/pmwaniki/vignette/tree/v1.0.1, and the associated Zenodo archive (10.5281/zenodo.17340120). The repository includes datasets, analysis scripts and documentation sufficient to reproduce the primary results and figures.
