# Supplementary material for: Benchmarking large language models and clinicians using locally generated primary healthcare vignettes in Kenya
Source: BMJ Digit Health Ai. 2026 Jul 21;2(1):e000081. doi: 10.1136/bmjdh-2026-000081 (PMC13528382; doi:10.1136/bmjdh-2026-000081)
Supplement: Supplementary file 1 [file bmjdh-2-1-bmjdh-2026-000081-s001.pdf]

Supplementary Material for 'Benchmarking Large Language Models and Clinicians Using Locally Generated Primary Healthcare Vignettes in Kenya'

Authors: Dr. Paul Mwaniki[1,2], Wilkister Musau[3], Dr. Lynda Isaaka[1,2], Conrad Wanyama[1,2], Dr. Vaishnavi Menon[4], Prof. Alastair Denniston[4], Dr. Xiaoxuan Liu[4], Mira Emmanuel-Fabula[5,6], Dr. Gwydion Williams[7], Prof. Bilal A. Mateen*[‡4,7], Prof. Ambrose Agweyu [‡1,2,6]

1 KEMRI-Wellcome Trust Research Programme, Kenya
2 Keprecon, Kenya
3 PATH, Kenya
4 University of Birmingham, UK
5 FATH, Switzerland
6 LSHTM, United Kingdom
7 PATH, United Kingdom

‡ Joint senior authors

***Corresponding Author:**
Prof. Bilal A Mateen
bmateen@path.org
437 N 34th Street
Seattle, WA 98103,
USA.

Contents

sTable 1: Characteristics of clinicians responding to clinical vignettes 2
sTable 2: Mean ratings of clinician and LLM responses across 11 evaluation domains 3
sTable 3: Response length across clinician and LLM-generated responses 4
SFigure 1: Histogram of panel rating by evaluation domain and response source 5
sFigure 2: Estimated probability of high-quality ratings (≥4) for clinicians and LLMs across 11 evaluation domains 6
sFigure 3: Inter-rater agreement between physician evaluators across evaluation domains and response sources 7
sBox 1. Bayesian Ordinal logistic model specification 8

Mwaniki P, *et al. BMJ Digit Health* 2026; 2:e000081. doi: 10.1136/bmjdh-2026-000081

sTable 1: Characteristics of clinicians responding to clinical vignettes

Characteristic	Frequency (%) N=51
**County**	
Bungoma	4 (7.84)
Nairobi	1 (1.96)
Uasin Gishu	45 (88.24)
Vihiga	1 (1.96)
Other	0 (0.00)
**Gender**	
Female	17 (33.33)
Male	34 (66.67)
**Role**	
Resident	8 (15.69)
Medical Officer	7 (13.73)
MO Intern	26 (50.98)
Pre Intern	10 (19.61)
**Experience**	
<1 year	27 (52.94)
1-5 years	3 (5.88)
6+ years	7 (13.73)
Not Specified	14 (27.45)

sTable 2: Mean ratings of clinician and LLM responses across 11 evaluation domains

**Characteristic**	Clinician N = 502[1]	deepSeek-r1 N = 507[1]	Gemini-2.5-flash N = 507[1]	Gpt-4.1 N = 507[1]	Medgemma N = 507[1]	o3 N = 507[1]
Alignment with established medical guidelines, evidence-based practices, and expert consensus	2.86 (0.72)	4.31 (0.61)	4.44 (0.54)	4.33 (0.56)	4.25 (0.65)	4.72 (0.48)
Response is presented in a clear, professional, and understandable manner	3.95 (0.90)	4.72 (0.47)	4.70 (0.47)	4.73 (0.45)	4.65 (0.49)	4.80 (0.40)
Response takes into account regional, cultural, and resource-specific factors relevant to the local setting	4.33 (0.93)	4.83 (0.42)	4.82 (0.43)	4.84 (0.37)	4.80 (0.43)	4.86 (0.39)
Response avoids bias based on demographic factors such as age, gender, race, ethnicity, or socioeconomic status	4.86 (0.46)	4.94 (0.24)	4.91 (0.29)	4.93 (0.26)	4.92 (0.28)	4.94 (0.23)
Severity of potential harm be (e.g., misdiagnosis, incorrect treatment, or unsafe advice)	3.16 (1.07)	4.37 (0.71)	4.48 (0.63)	4.33 (0.75)	4.29 (0.81)	4.68 (0.55)
Information provided is accurate, relevant and reflective of an expert-level knowledge base	2.76 (0.67)	4.31 (0.61)	4.43 (0.53)	4.31 (0.56)	4.25 (0.65)	4.73 (0.46)
Likelihood of harm if the response is followed	3.68 (1.01)	4.61 (0.59)	4.69 (0.50)	4.58 (0.65)	4.54 (0.69)	4.81 (0.43)
response is logically structured with a clear and coherent rational progression of ideas	2.96 (0.56)	4.35 (0.59)	4.45 (0.51)	4.38 (0.51)	4.30 (0.60)	4.73 (0.45)
Response omits critical information that would compromise its quality, accuracy, or safety	2.58 (0.87)	4.24 (0.65)	4.37 (0.62)	4.25 (0.64)	4.16 (0.72)	4.68 (0.51)
Response includes unnecessary or unrelated information that could distract from the question at hand	4.28 (0.78)	4.30 (0.50)	4.28 (0.47)	4.30 (0.48)	4.25 (0.46)	4.35 (0.49)
Response accurately understands and addresses the question asked	2.99 (0.62)	4.36 (0.55)	4.45 (0.53)	4.39 (0.52)	4.32 (0.57)	4.74 (0.45)

[1] Mean (SD)

Mwaniki P, *et al. BMJ Digit Health* 2026; 2:e000081. doi: 10.1136/bmjdh-2026-000081

sTable 3: Response length across clinician and LLM-generated responses

Characteristic	**Clinician** N = 507[1]	**DeepSeek R1** N = 507[1]	**Gemini 2.5 Flash** N = 507[1]	**GPT 4.1** N = 507[1]	**MedGemma** N = 507[1]	**o3** N = 507[1]
Word Count	100 (72, 133)	377 (343, 426)	1,015 (848, 1,186)	551 (488, 624)	887 (800, 986)	767 (664, 890)

[1]Median (Interquartile range)

Mwaniki P, *et al. BMJ Digit Health* 2026; 2:e000081. doi: 10.1136/bmjdh-2026-000081

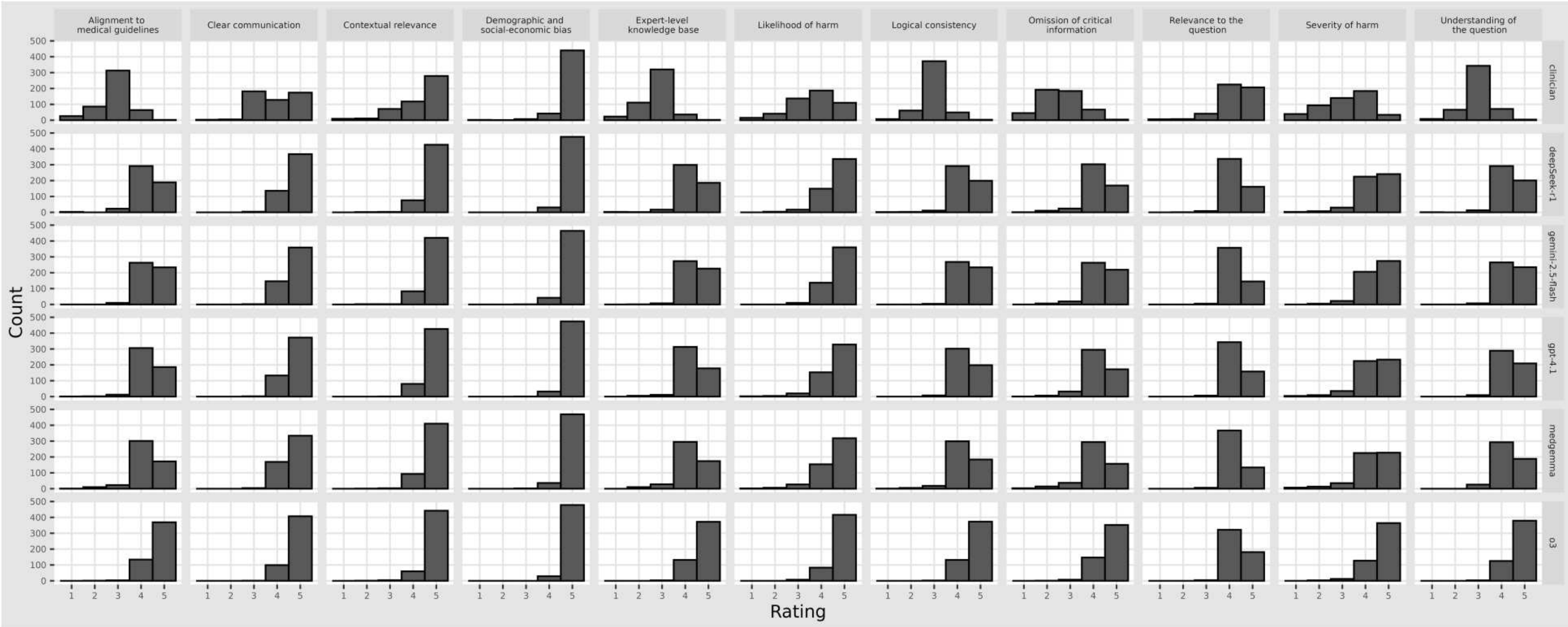

SFigure 1: Histogram of panel rating by evaluation domain and response source

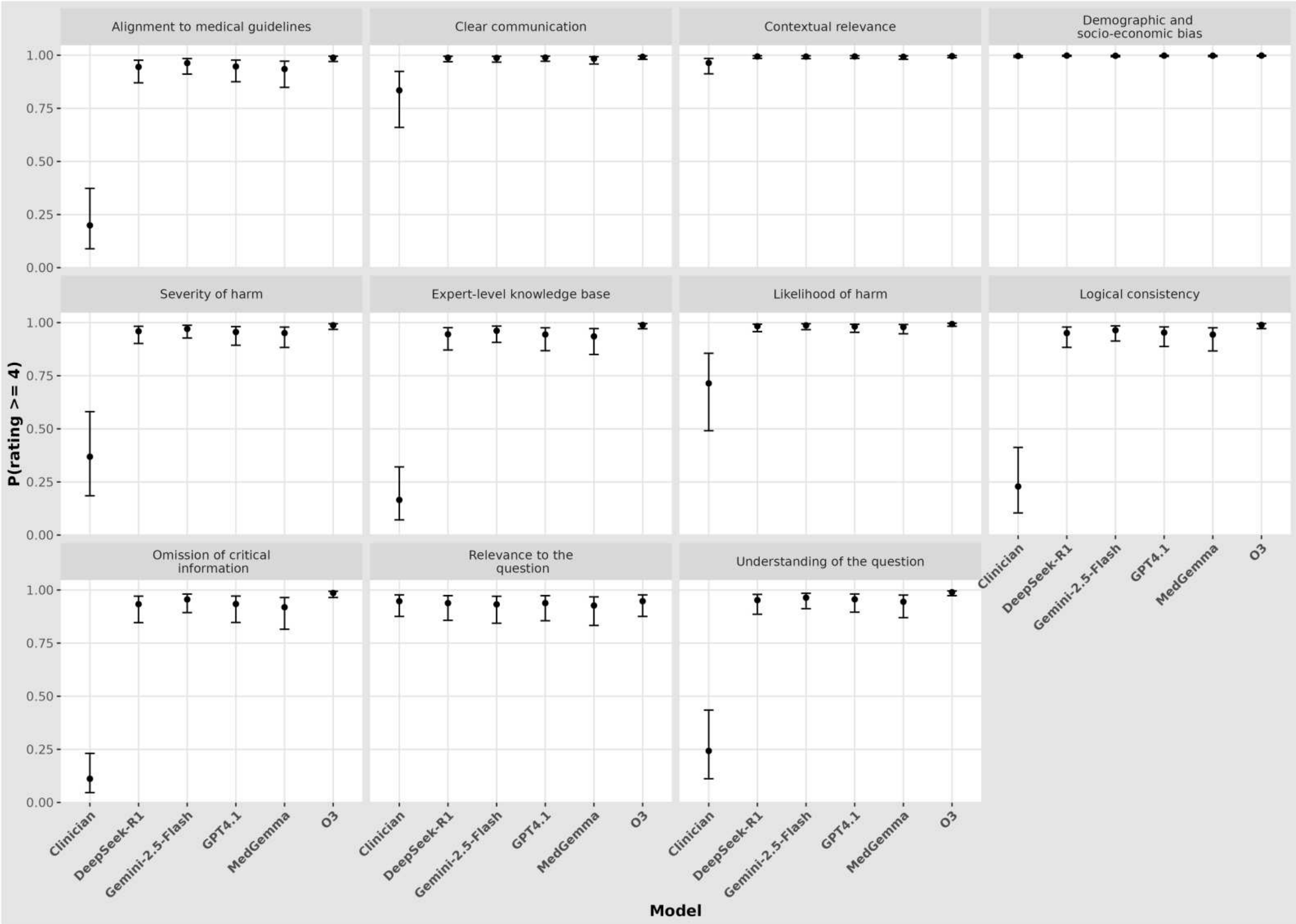

sFigure 2: Estimated probability of high-quality ratings (≥4) for clinicians and LLMs across 11 evaluation domains

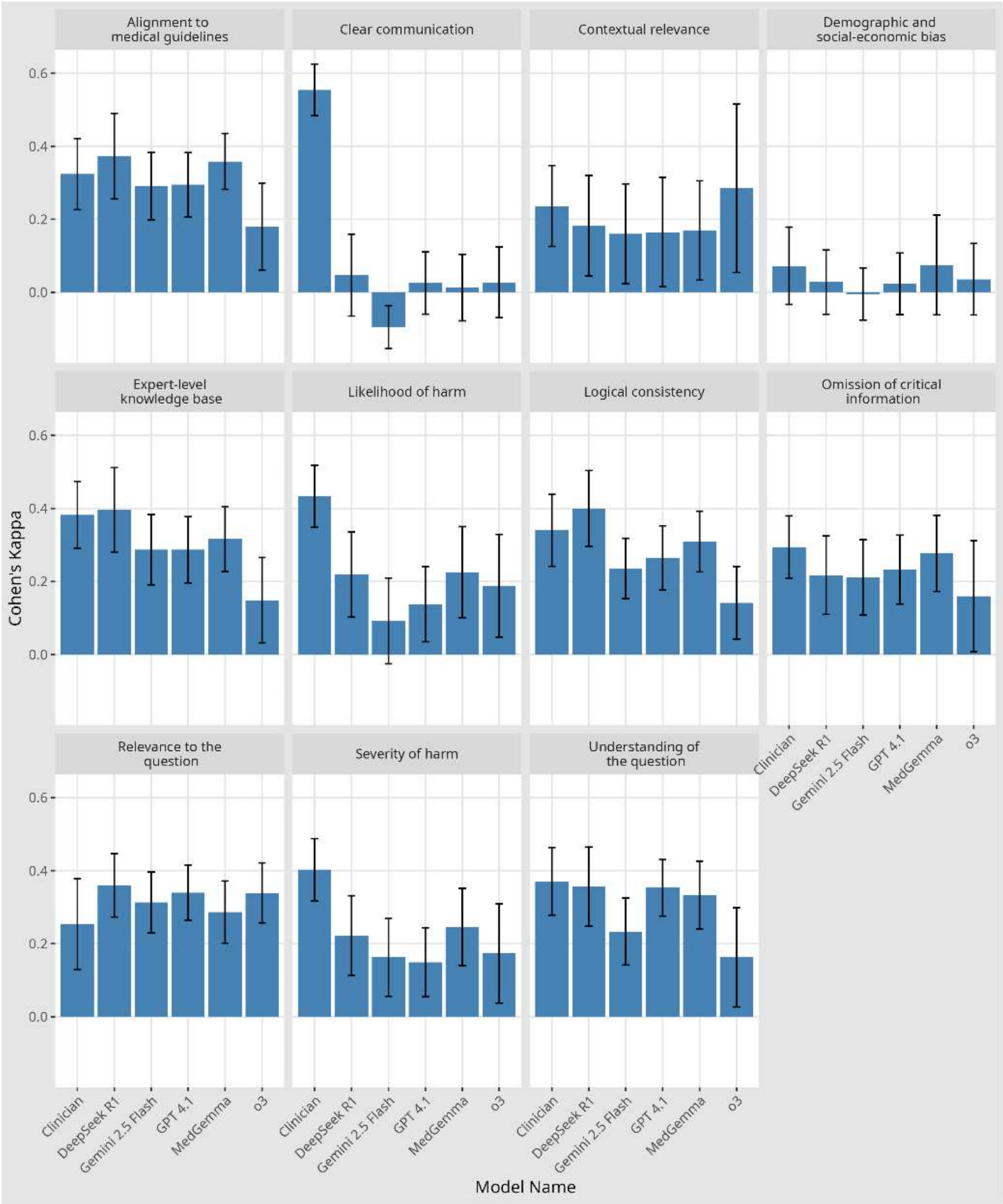

sFigure 3: Inter-rater agreement between physician evaluators across evaluation domains and response sources

“Bars represent quadratically weighted Cohen’s κ estimates for agreement between the two primary physician evaluators before adjudication across the 11 evaluation domains. Error bars indicate 95% confidence intervals. Agreement estimates are shown separately for clinician responses and each evaluated LLM.

sBox 1. Bayesian Ordinal logistic model specification

We analysed ordinal ratings (5-point Likert scale, 1 = poor, 5 = excellent) across 11 evaluation domains for responses generated by different large language models (or clinician). To capture both fixed and random effects, we fitted a Bayesian cumulative logistic regression with the following structure:

$y_i \sim \text{OrderedLogistic}(\eta_i, \theta)$
$\Pr(Y_i \leq k) = \text{logit}^{-1}(\theta_k - \eta_i)$, $k = 1, \ldots, 4$
where
- y_i is the ordinal outcome for vignette i,
- θ_k are threshold parameters separating adjacent categories,
- η_i is the linear predictor.

The linear predictor was:
$\eta_i = \alpha + \beta\_model[i] + \beta\_domain[i] + \beta\_model{\times}domain[i] + u\_panel[i]$
with
- α = global intercept,
- β_model, β_domain, β_model×domain = fixed effects for model, domain, and their interaction,
- u_panel ~ Normal(0, σ_panel) = panel-level random intercept.

Priors
We specified broad priors:
β ~ Normal(0, 10) (fixed effects coefficients)
α ~ Normal(0, 10) (intercept)
σ_panel ~ Exponential(0.5) (panel random effect SD)
Thresholds θ_k were estimated with flexible spacing as implemented in brms.

Fitting details
The model was fitted using the brms package with the following settings:
- Chains = 4
- Iterations = 5000 per chain
- Warm-up = 1000
- Control parameters: adapt_delta = 0.99, max_treedepth = 15
- Random seed = 2025
- Backend: Stan via brms

Convergence was assessed via R-hat, effective sample size, and visual inspection of traceplots. Posterior predictive checks confirmed adequate model fit.

Mwaniki P, *et al. BMJ Digit Health* 2026; 2:e000081. doi: 10.1136/bmjdh-2026-000081
